# Examining second-stage shelters: insights into housing instability and tailored support for IPV survivors

**DOI:** 10.1186/s12889-023-17623-2

**Published:** 2024-02-07

**Authors:** Ebony Rempel, Lorie Donelle, Jodi Hall, Nadine Wathen

**Affiliations:** 1 Faculty of Health Sciences, Western University, London, ON N6A 5B9 Canada; 2 College of Nursing, University of South Carolina, 1601 Greene St., Columbia, SC 29208 Canada; 3 Department of Nursing, Fanshawe College, London, ON Canada

**Keywords:** Women, Intimate-partner violence, Violence against women, Transitional shelter, Second-stage shelter, Housing

## Abstract

**Background:**

Intimate Partner Violence (IPV) exposes women and children to a wide range of challenges across housing, employment, social connections, and child well-being and is a public health issue. IPV survivors are at heightened risk of housing insecurity and homelessness. Emergency shelters have historically offered respite and support, but the emergence of second-stage shelters provides longer-term solutions. Despite their significance, there has been a lack of comprehensive research on second-stage shelters. This study focuses on understanding the needs of IPV survivors accessing second-stage shelters, aiming to illuminate unexplored aspects of support. To examine the current published peer-reviewed literature and gray literature on second-stage shelters, a scoping review was conducted.

**Methods:**

This scoping review used the method suggested by Arksey & O’Malley (2005) and considered all studies that focused on women who had experienced IPV and were accessing transitional housing/second-stage shelters.

**Results:**

Sixteen articles, mainly from the USA and published between 1985 and 2022, were included in the analysis. The findings highlighted themes of (1) a safe(r) place, with the subtheme of ‘gated’ communities, and (2) programming and services, with the subtheme of does one size fit all? and (3) insider support, with subthemes of paid insider support and peer insider support.

**Conclusions:**

Housing instability was evident, and the need for multiple and individualized tailored options of programming and support along with housing security was identified. Second-stage housing policy and practice implications are addressed which illuminate unexplored aspects of support.

## Background

Survivors of intimate partner violence (IPV) seek assistance across various domains, including housing, employment, social connections, and children’s well-being. Factors influencing these areas differ for each individual [1]. Women who have experienced IPV generally seek help from formal (service providers, agencies, organizations) and informal (friends, family) channels [2, 3]. IPV creates a cascade of negative consequences for women that includes the need for ongoing information seeking and problem solving to manage and mitigate the devastating effects on health, social, and family functioning, as well as a depletion of economic resources and community support [4, 5]. Although survivors typically turn to their interpersonal networks first for information, the nature of abuse often restricts supportive relationships [6, 7]. For many women experiencing IPV, accessing information and services is complex due to structural barriers and the stigma attached to being a victim, yet women require an intensity of informal and formal resources to cope with the widespread consequences of IPV.

IPV survivors are more likely to experience housing insecurity or homelessness than those who have not experienced IPV. Community social service providers have responded, in part, to IPV through the development of community-based emergency shelters designed for women and their children that include access to support and services such as childcare, outreach services, and public education [8]. Women’s shelters have played a vital role since the 1970s, serving as important access points for information, services, and support for women in need [9]. Emergency shelters are intended as places of temporary respite and accommodation for women and children experiencing family violence [8, 10, 11].

Beyond emergency shelters, second-stage shelters, also called transitional shelters/houses, or interval shelters/houses, were constructed to accommodate the longer-term housing and transitional needs of women and their children. Different from emergency shelter services, second-stage shelters are a form of transitional housing for survivorsof domestic violence / IPV. Second-stage shelters provide longer extended housing accommodationsto women and children and can consist of apartment type units in one facility with some common areas or individual units dispersed among different buildings but within the same neighbourhood or geographical location. While there are many terms used in the literature to reflect longer-term supportive housing for women who have experienced IPV, the term *second-stage shelters* will be used in this scoping review in reference to this type of accommodation. Second-stage shelters vary with respect to service delivery, services available, and length of stay [8, 12] but are usually community-based facilities similar to apartment-type units with support services intended to support the needs of women and their children who have experienced IPV. In general, second-stage shelters provide longer-term accommodation, from six months up to 24 months, and women access second-stage shelters in multiple ways, most commonly through a referral from an emergency shelter or an individual application [13]. Different from emergency shelters, women in second-stage shelters will often pay rent and are responsible for maintaining their rental unit and for their basic needs, such as food. Given that most second-stage shelters began operating in the 1980s, with the most recent published research in 1985, little is known about second-stage shelters. Therefore, the focus of this research is to investigate the information, service, and support needs of women who had experienced IPV and were accessing transitional housing/second-stage shelters.

## Methods

A scoping review is an appropriate way to describe literature and other sources of information that include a range of different study designs and methods, especially when a topic is complex and has not been comprehensively reviewed. This scoping review used the method suggested by Arksey & O’Malley (2005) and considered all studies that focused on women who have experienced IPV and were accessing transitional housing/second-stage shelters. The framework for scoping reviews and the PRISMA-ScR (Preferred Reporting Items for Systematic reviews and Meta-Analyses extension for Scoping Reviews) checklist were used to report findings [14].

### Inclusion criteria

Included in this review were peer-reviewed articles and gray literature published in English that focused on the information, service, and support needs of women who had experienced IPV and accessed second-stage shelters globally. Articles published from January 1, 1980, to January 29, 2023, were eligible for inclusion in the review.

### Exclusion criteria

We excluded opinion articles, letters to the editor, response letters, dissertations, and protocol papers. Articles that focused primarily on homelessness without the context of IPV were excluded, as were those with a primary focus on veterans and sex workers. Articles that focused only on emergency shelters were not included. Articles that were not published in English were excluded.

### Stage one– identifying the research question

There exists a broad range of legal, health-related, and community support for women experiencing IPV; however, evidence on women’s experiences regarding second-stage housing is limited. Decisions by women to seek support require interaction with multiple social systems (legal, income, housing, etc.) that are embedded with complex power inequalities that create and sustain the conditions for violence against women [15–17]. The specific research question is as follows: What research is available on women’s experiences of accessing and residing in violence against women (VAW) second-stage shelters?

### Stage two– identifying relevant studies

Social Services Abstracts, Social Work Abstracts, Library Literature and Information Science Full Text, Library Information Science and Technology Abstracts, Medline, Scopus, CINAHL, and sociological abstracts were searched using the database-specific indexing terms outlined in Table 1. Using these same search terms, the researcher searched Statistics Canada, World Health Organization, Women’s Shelters Canada, and Status of Women Canada websites for gray literature.

**Table 1:** Search strategy for electronic databases

Database	Search strategy
Social Services Abstracts	noft("second-stage shelter" OR "transitional housing" OR "transitional shelter" OR "second-stage housing") AND noft(women)
Social Work Abstracts	("second-stage shelter" OR "transitional housing" OR "transitional shelter" OR "second-stage housing") AND (women)
Library Literature & Information Science Full Text (H.W. Wilson)	("second-stage shelter" OR "transitional housing" OR "transitional shelter" OR "second-stage housing") AND (women)
Library, Information Science & Technology Abstracts (LISTA)	("second-stage shelter" OR "transitional housing" OR "transitional shelter" OR "second-stage housing") AND (women)
MEDLINE (Ovid)	(("second-stage shelter" or "transitional housing" or "transitional shelter" or "second-stage housing") and women).af.
Scopus	(TITLE-ABS-KEY ("second-stage shelter") OR TITLE-ABS-KEY ("transitional housing") OR TITLE-ABS-KEY ("transitional shelter") OR TITLE-ABS-KEY ("second-stage housing") AND TITLE-ABS-KEY (women))
CINAHL	"second-stage shelter" OR "transitional housing" OR "transitional shelter" OR "second-stage housing" AND women"
Sociological Abstracts	noft("second-stage shelter") OR noft("transitional housing") OR noft("transitional shelter") OR noft("second-stage housing") AND noft(women)

### Stage three– study selection

**Fig. 1:**
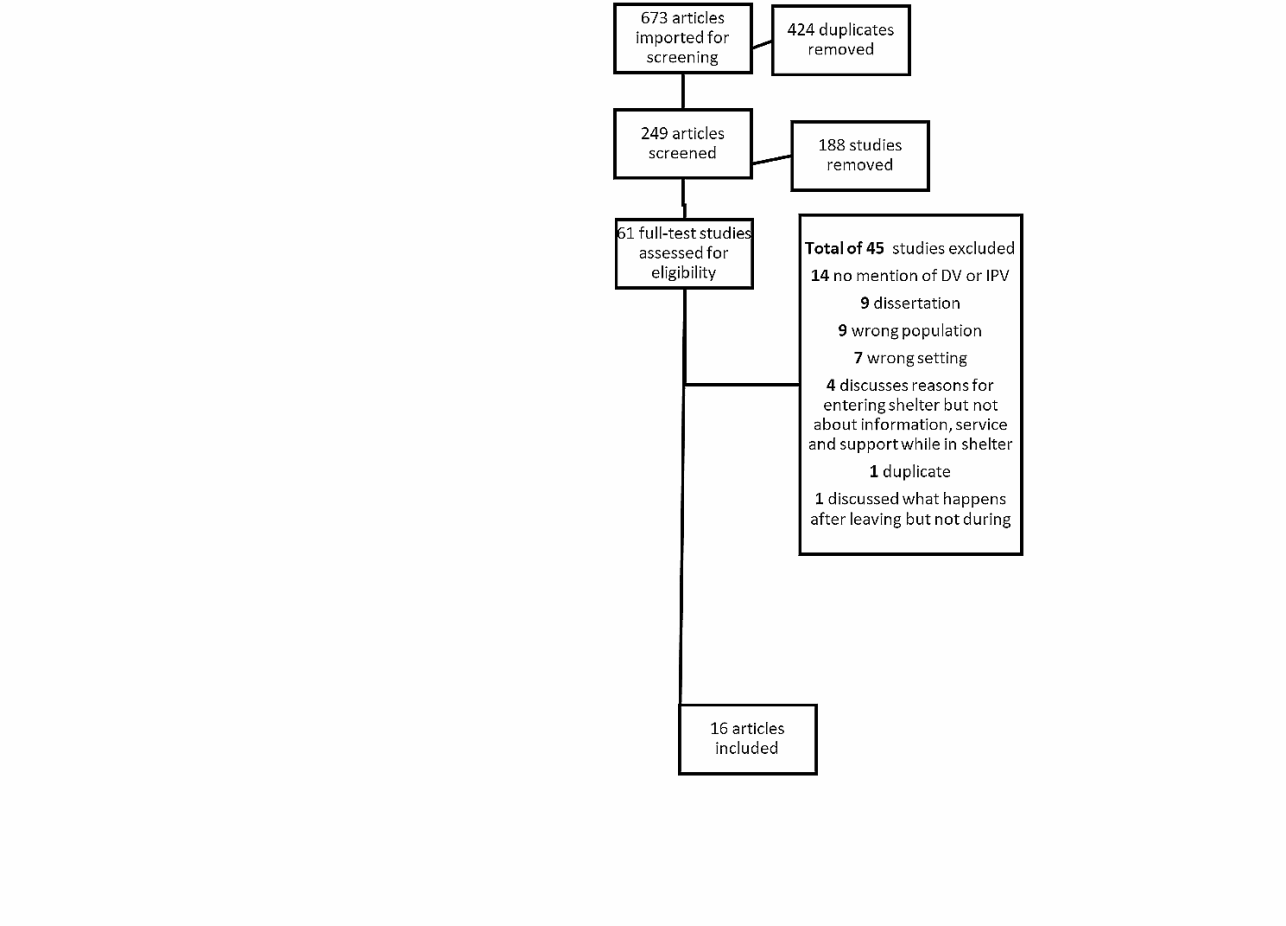
PRISMA flow chart of the identification process

The study selection process involved a systematic sorting of the literature using inclusion/exclusion criteria. Covidence, an online research platform, was utilized to organize the retrieved articles. Initially, 673 articles were obtained through the database search, and after removing duplicates, 249 articles remained. Two independent reviewers examined the titles and abstracts, eliminating irrelevant articles. After a thorough review, 188 articles did not meet the inclusion criteria and were excluded. The remaining 61 articles were read in full, and any that did not meet the criteria were removed. The PRISMA flow chart in Fig. 1 illustrates the identification and selection process for the review.

### Stage four– charting the data

The scoping review included a total of sixteen articles, as shown in Table 2. Twelve articles were published in the USA, three in Canada, and one in Australia. The publication dates ranged from 1985 to 2022. Among the included articles, eleven used a qualitative research design, two employed a mixed methods approach, and two were quantitative studies. Twelve articles presented the perspectives of survivors through interviews or focus groups, involving a total of 322 survivors across all studies. Two articles focused on reviewing and synthesizing literature, policies, and data. Additionally, four articles incorporated the viewpoints of shelter staff and service providers through surveys, focus groups, and interviews.

**Table 2:** Articles included in the scoping review according to the date of publication

	Title	Reference	Location	Study Type
1	Transitional housing planning and design: Practice and education by women for women in the USAA	(Sprague, 1985)	USA	Qualitative
2	Moving along: An exploratory study of homeless women with children using a transitional housing program	(Fogel, 1997)	USA	Qualitative
3	Transitional supportive housing programs: Battered women’s perspectives and recommendations	(Melbin et al., 2003)	USA	Qualitative
4	Sleep of children of abused women in transitional housing	(Humphreys & Lee, 2005)	USA	Quantitative
5	A descriptive analysis of transitional housing programs for survivors of intimate partner violence in the United States	(Baker et al., 2009)	USA	Mixed Methods
6	Domestic violence, housing instability, and homelessness: A review of housing policies and program practices for meeting the needs of survivors	(Baker et al., 2010)	USA	Qualitative
7	Aboriginal women’s perceptions and experiences of a family violence transitional accommodation service	(Wendt & Baker, 2013)	Australia	Qualitative
8	'A place to rest’: The role of transitional housing in ending homelessness for women in Calgary, Canada	(Fotheringham et al., 2014)	Canada	Qualitative
9	Navigating homelessness and navigating abuse: How homeless mothers find transitional housing while managing intimate partner violence	(Long, 2015)	USA	Qualitative
10	Building a novel health curriculum for survivors of intimate partner violence residing at a transitional housing program	(Ragavan et al., 2018)	USA	Qualitative
11	Examining the needs and experiences of domestic violence survivors in transitional housing	(Clark et al., 2019)	USA	Qualitative
12	Breaking the cycle of abuse and closing the housing gap: Second stage shelters in Canada	(Maki, 2020)	Canada	Mixed Methods
13	Playing by the rules: Agency policy and procedure in service experience of IPV survivors	(Wood et al., 2020)	USA	Qualitative
14	Centering our voices: Experiences of violence among homeless African American women	(Deal et al., 2022)	USA	Qualitative
15	Outcomes associated with participation in a sober living home for women with histories of domestic and sexual violence victimization and substance use disorders	(Edwards et al., 2022)	USA	Qualitative
16	Housing interventions for women experiencing intimate partner violence: A systematic review	(Yakubovich et al., 2022)	Canada	Quantitative

### Stage five– collate, summarize, and report results

Thematic findings are described in detail below and include: (1) A Safe(r) Place, with the subtheme of ‘Gated’ Communities; (2) Programming and Services, with the subtheme of Does One Size Fit All? and (3) Insider Support, with subthemes of Paid Insider Support and Peer Insider Support.

## Results

### A safe(r) place

Safety is a paramount concern in the existence of women’s shelters, as leaving violent relationships puts women at risk of femicide [5]. Most of the research highlighted in this scoping review emphasized second-stage shelters as a safe place for women and their children to stay and pointed to the importance of providing that safety [13, 18–24]. Housing instability and lack of affordable, safe housing options for women leaving second-stage shelters was evident, and the need for multiple solutions with tailored choices and options for safe housing was identified.

### ‘Gated’ communities

Second-stage shelters are a unique type of longer-term transitional housing for survivors of violence. Articles from this scoping review reported on the barriers to accessing second-stage shelters, such as the organizational policies (e.g., curfews, restrictions on visitors) that made it difficult to live within the spaces [13, 18–26]. While some second-stage shelters adopted low-barrier and harm reduction practices [13], instances of ongoing substance use and mental health difficulties meant that women were likely denied access to a second-stage shelter [13, 18, 23, 25, 27, 28].

Many second-stage shelters have rigid ‘house’ rules (e.g., curfews, restrictions on substance use, mandatory check-ins, restrictions on visitors including adolescent male sons) and negative consequences for those who do not abide by them. While the intention of the ‘house’ rules is to ensure individuals’ safety and security, there is evidence that women experienced the rules as invasive at times (e.g., sharing many details of your life in order to access shelter spaces) and isolating (e.g., restrictions on visitors) [19, 20, 22, 24, 25]. Second-stage shelter residents reported that the ‘house’ rules set for acceptable resident behaviors (e.g., curfews, personal surveillance) also had the unintended consequence of making them feel trapped and controlled, reminiscent of the experiences they had within their IPV relationship [24].

Access to second-stage shelters appeared inequitable in that access was notthe same for all groups of people. Disabled women, 2SLGBTQ women, Indigenous women, women of color, and new immigrant women encountered additional obstacles in accessing second-stage shelters. Cultural needs, accessibility, and language barriers posed challenges, as not all spaces accommodated these diverse requirements [19, 21, 26, 29, 30]. The protective measures implemented to ensure safety inadvertently created difficulties for certain groups of women in accessing shelter services. Of the sixteen articles included in this scoping review, only six commented on the inequities and potential barriers to accessing second-stage shelter spaces among marginalized groups.

### Programming and services

Second-stage shelters host workshops and information sessions on a variety of topics (e.g., finances, nutrition, parenting), which assume that these are the types of support women require. Articles in this review discussed the importance of providing individualized case management support targeted to what survivors wanted [13, 18, 20, 22, 24, 25, 30]. Supportive educational programming was made available by many second-stage shelters related to job/educational assistance, legal services, childcare support, housing support, counseling, transportation, referrals to other agencies, safety planning, food and food programs, and parenting classes [13, 18, 29, 30, 19–23, 26–28]. Second-stage shelters provided specialized services, including survivor-centered programming with expertise in gender-based violence, availability of counseling services, and safety planning [13, 28].

### Does one size fit all?

Some researchers were critical of the mandatory nature of programming that still exists within shelter systems and was almost always identified by women as a barrier [13, 21, 23–25, 29]. Not all women accessing second-stage shelters require the same type of support or information, and some groups, such as Indigenous peoples [26] and African American women [29], require unique and tailored programming. For example, Melbin and colleagues (2003), who interviewed second-stage shelter support staff and residents, advised those working in shelters not to assume that all women need basic skill-building and extensive case management. Clark and colleagues (2019) discovered that some groups (e.g., those in severe danger, immigrant survivors, and those with greater psychosocial needs) appreciated intense programming and safety protocols, yet others appreciated a less structured model of shelter living. There was a lack of overall diversity noted within the literature from this scoping review, and not all articles attended to the different types of support diverse women will require while accessing second-stage shelters.

### Insider support

A trauma-informed model that included elements of peer support and peer debriefing was helpful within second-stage shelter spaces [21, 24]. While much of the programming was formalized, insights from shelter staff and women residing within the spaces highlighted the importance of information access shared through informal relationships established among women residing within the shelter [20–22, 24]. Fogel (1997) found that relationship development within second-stage shelter support groups was an important component for healing. A community of women to connect with that had similar experiences was valued [21]. Women who experienced coercion, control, and isolation in the context of their abusive relationship also tended to have negligible networks of informal support, and insider support from other residents and/or staff was important for women accessing second-stage shelters [13, 21, 26].

### Paid insider support

All articles included in this review on second-stage shelters reported that paid staff were available to support women. There were tensions that came with this support that made women residing in second-stage shelters feel disrespected at times [21]. The established organizational boundaries created strain due to the juxtaposition between having to enforce shelter policies and being a supportive listener [21, 26]. Women accessing one second-stage shelter program indicated that it was difficult to develop a strong relationship with the program staff [30], as having multiple staff present according to their scheduled work shifts made it difficult to develop a foundation of trust. However, Melbin et al. (2003) found that women identified the staff of second-stage shelters as allied advocates [23], especially when there was choice and variability involved in terms of available programming. Additional research is warranted to determine whether the mandatory nature of educational programming that was flexible and attentive to women’s priority information, service, and support needs would be perceived as helpful rather than generically prescriptive [13, 21, 26].

### Peer insider support

Women residing in second-stage shelters reported that having a community of women to share their experiences with had a positive influence on their lives [21]. Women who were able to make decisions independently felt better supported when they were able to share their experiences with a community of women [13, 21]. These relationships women created with other shelter residents made their stay at the shelter easier [19, 26, 27]. Maki (2020) noted that building new friendships and networks of support was critical for women in their healing journey.

## Discussion and public health implications

This review of the literature focused on women’s experiences in accessing and residing in VAW second-stage shelters. Most of the research in this review was conducted in high-income countries, particularly the USA. However, further research is needed to understand the existence and operations of second-stage shelters in a wider range of countries, especially low- and middle-income contexts [28]. Accessing support in areas such as housing, employment, social connections, and child well-being is complex [1] and can deplete personal resources and social support networks [4, 10, 31]. Comprehensive and sustainable funding is a challenge for recruiting and retaining qualified staff who can effectively support women in accessing necessary resources [13]. Furthermore, the disconnect between the government [13], the homeless sector [25], and VAW shelters creates funding challenges and a lack of collaboration in the delivery of direct services.

Our findings highlight the importance of second-stage shelters as temporary safe spaces for women and their children, particularly for those facing financial strain and homelessness. However, as indicated in the findings, access to second-stage shelters remains inequitable, with inadequate availability of shelter units, especially in rural, remote, and Indigenous communities. Increased collaboration with gender diverse, racialized, and Indigenous communities is crucial to understanding and addressing the unique experiences the different groups of women who access second-stage shelters [13, 25, 26]. Equity-oriented policy review offers an important strategy to address equity of access to shelter spaces.

While most or all second-stage shelters provide supportive and educational programs, the review of the literature emphasized the need for a diverse range of programming and services. Women value flexibility in topics and delivery formats, while mandatory programming is often perceived as unsupportive. Voluntary services and optional participation in services and programming provide women with choice. Small and manageable programming optionsthat are based on the belief that survivors have the ability to make decisions about their lives and can be individualized or group-based based on women’s choices and needs are crucial [13, 24–26]. Not all women require extensive case management or security measures, and collaboration with the homelessness sector can provide alternative options, such as rapid rehousing [13, 19, 23–26]. Limited long-term housing options can force women to focus on shelter requirements rather than building resources for their future [22].

It is important to note that articles not published in English were excluded, leading to language bias and potential exclusion of relevant studies. Additionally, scoping reviews do not assess the quality of the literature but aim to identify gaps [14]. Therefore, the conclusions of this review are based on summarizing the results and do not include an assessment of study quality.

In summary, this scoping review highlights barriers to accessing second-stage shelters, including restrictive rules and programming, while also acknowledging positive aspects related to safety, tailored programming, and community building. There is a pressing need for safe and affordable long-term housing for women and children who have experienced IPV. Governments and funders must consider the negative aspects of second-stage shelters and reduce restrictions tied to mandated programming that may not suit all women and families.

## Data Availability

The datasets used and/or analysed during the current study are available from the corresponding author on reasonable request.

## References

[1] Goodman LA, Thomas K, Cattaneo LB (2016). Survivor-defined practice in domestic violence work: Measure development and preliminary evidence of link to empowerment. J Interpers Violence.

[2] Evans MA, Feder GS (2016). Help-seeking amongst women survivors of domestic violence: A qualitative study of pathways towards formal and informal support. Heal Expect.

[3] Westbrook L, Gonzalez ME (2011). Information Support for Survivors of Intimate Partner Violence: Public Librarianship’s role. Public Libr Q.

[4] Brownridge DA (2006). Violence against women post-separation. Aggress Violent Behav.

[5] World Health Organization, WHO. Understanding and addressing violence against women. Epub ahead of print 2012. 10.2307/1319341.

[6] Lyons M, Brewer G (2022). Experiences of intimate Partner violence during Lockdown and the COVID-19 pandemic. J Fam Violence.

[7] Mojahed A, Brym S, Hense H, et al. Rapid Review on the associations of Social and geographical isolation and intimate Partner violence: Implications for the Ongoing COVID-19 pandemic. Front Psychiatry. 2021;12.Epub ahead of print. 10.3389/fpsyt.2021.578150 10.3389/fpsyt.2021.578150 PMC8076499 33927649

[8] Maki K (2019). Transitioning to a Life Free from Violence: Second Stage shelters in Canada.

[9] Goodhand M (2017). Runaway wives and rogue feminists: The origins of the women’s shelter mmovement in Canada.

[10] Beattie S, Hutchins H. Shelters for abused women in Canada, https://www.statcan.gc.ca/pub/85-002-x/2015001/article/14207-eng.htm (2015).

[11] Galano MM, Hunter EC, Howell KH (2013). Predicting Shelter Residence in Women Experiencing recent intimate Partner violence. Violence against Women.

[12] Barton S, Hungler K, McBride D et al. Alberta Research Project Report for Provincial Stakeholders: Rural and Northern Community Response to Intimate Partner Violence. 2015.

[13] Maki K. Breaking the Cycle of Abuse and Closing the Housing Gap: Second Stage Shelters in Canada. Ottawa, ON, 2020.

[14] Arksey H, O’Malley L (2005). Scoping studies: Towards a methodological framework. Int J Soc Res Methodol.

[15] Edwards KM, Mattingly MJ, Dixon KJ (2014). Community matters: Intimate partner violence among rural young adults. Am J Community Psychol.

[16] Hughes J, Chau S, Vokrri L (2016). Mothers’ narratives of their involvement with Child Welfare services. Affilia.

[17] Ragusa AT (2012). Rural Australian women’s legal help seeking for intimate Partner violence: Women intimate Partner violence victim survivors’ perceptions of criminal justice support services. J Interpers Violence.

[18] Baker CK, Niolon PH, Oliphant H (2009). A descriptive analysis of transitional housing programs for survivors of intimate partner violence in the United States. Violence against Women.

[19] Clark DL, Wood L, Sullivan CM (2019). Examining the needs and experiences of domestic violence survivors in transitional housing. J Fam Violence.

[20] Fogel SJ (1997). Moving along: an exploratory study of Homeless Women with Children using a Transitional Housing Program. J Sociol Soc Welf.

[21] Fotheringham S, Walsh CA, Burrowes A (2014). A place to rest’: The role of transitional housing in ending homelessness for women in Calgary, Canada. Gender. Place Cult.

[22] Long SM (2015). Navigating homelessness and navigating abuse: How homeless mothers find transitional housing while managing intimate partner violence. J Community Psychol.

[23] Melbin A, Sullivan CM, Cain D (2003). Transitional Supportive Housing Programs: Battered women’s perspectives and recommendations. Affil - J Women Soc Work.

[24] Wood L, Cook Heffron L, Voyles M (2020). Playing by the rules: Agency Policy and Procedure in Service Experience of IPV survivors. J Interpers Violence.

[25] Baker CK, Billhardt KA, Warren J (2010). Domestic violence, housing instability, and homelessness: A review of housing policies and program practices for meeting the needs of survivors. Aggress Violent Behav.

[26] Wendt S, Baker J (2013). Aboriginal Women’s perceptions and experiences of a Family Violence Transitional Accommodation Service. Aust Soc Work.

[27] Edwards KM, Wheeler L, Siller L et al. Outcomes Associated with participation in a Sober Living Home for Women with histories of domestic and sexual violence victimization and Substance Use disorders. Traumatology (Tallahass Fla). Epub ahead of print 2022. 10.1037/trm0000394.

[28] Yakubovich AR, Bartsch A, Metheny N (2022). Housing interventions for women experiencing intimate partner violence: A systematic review. Lancet Public Heal.

[29] Deal E, Hawkins M, Del Carmen Graf M et al. Centering Our Voices: Experiences of Violence Among Homeless African American Women. Violence Against Women. Epub ahead of print 2022. 10.1177/10778012221117599. 10.1177/10778012221117599 36017557

[30] Ragavan M, Bruce J, Bair-Merritt M (2018). Building a Novel Health Curriculum for survivors of intimate Partner Violence residing at a Transitional Housing Program. Violence against Women.

[31] Wilson KS, Silberberg MR, Brown AJ (2007). Health needs and barriers to healthcare of women who have experienced intimate partner violence. J Womens Health (Larchmt).

